# The Value of Clinical Prediction Models in General Practice: A Qualitative Study Exploring the Perspectives of People With Lived Experience of Depression and General Practitioners

**DOI:** 10.1111/hex.70059

**Published:** 2024-12-18

**Authors:** Andrew S. Moriarty, Joanne Castleton, Dean McMillan, Richard D. Riley, Kym I. E. Snell, Lucinda Archer, Lewis W. Paton, Simon Gilbody, Carolyn A. Chew‐Graham

**Affiliations:** ^1^ Department of Health Sciences, Hull York Medical School University of York York UK; ^2^ School of Medicine Keele University Keele UK; ^3^ Member of Patient Advisory Group University of York York UK; ^4^ Institute of Applied Health Research University of Birmingham and National Institute for Health and Care Research (NIHR) Birmingham Biomedical Research Centre Birmingham UK

**Keywords:** depression, diagnosis, general practice, prediction model, primary care, prognosis

## Abstract

**Introduction:**

Prediction models are increasingly being used to guide clinical decision making in primary care. There is a lack of evidence exploring the views of patients and general practitioners (GPs) in primary care around their use and implementation. We aimed to better understand the perspectives of GPs and people with lived experience of depression around the use of prediction models and communication of risk in primary care.

**Methods:**

Qualitative methods were used. Data were generated over 6 months (April to October 2022) through semi‐structured interviews with 23 people with lived experience of depression and 22 GPs. A multidisciplinary research team and Patient Advisory Group were involved throughout the study. Data were analysed inductively using thematic analysis.

**Results:**

GPs describe using prediction models in consultations only when the models are either perceived to be useful (e.g., because they help address an important clinical problem) or if GPs feel compelled to use them to meet financial or contractual targets. These two situations are not mutually exclusive, but if neither criterion is met, a model is unlikely to be used in practice. People with lived experience of depression and GPs reported that communication of model outputs should involve a combination of risk categories, numerical information and visualisations, with discussions being tailored to the individual patients involved. Risk prediction in a mental health context was perceived to be more challenging than for physical health conditions.

**Conclusion:**

Clinical prediction models are used in practice but thought must be given at the study development stage to how results will be presented and discussed with patients. Meaningful, embedded public and patient involvement and engagement are recommended when developing or implementing clinical prediction models.

**Patient or Public Contribution:**

We used a combination of embedded consultation and collaboration/co‐production in our approach to public and patient involvement in this study. A Patient Advisory Group made up of people with lived experience of depression were involved from study conception and contributed to study design, participant recruitment, interpretation of findings and dissemination (including in the preparation of this manuscript).

## 1 Introduction

Clinical prediction models are tools that use information about multiple predictor variables to estimate an individual's risk of either current (diagnosis) or future (prognosis) outcomes. Prediction models can produce various kinds of outputs, including an individual's estimated risk (probability) of an outcome or a corresponding category/class membership (e.g., high‐, medium‐ or low‐risk category). They can be designed to provide instruction about what should happen next based on their predictions (clinical decision rules) or they can provide information to guide discussions, patient counselling and shared decision making (decision aids) [1].

Prediction models are increasingly being used to facilitate a personalised medicine approach in both primary care settings [2] and mental health contexts [3]. There have been few qualitative studies exploring clinician and patient perspectives of clinical prediction models in practice. Only one qualitative study has explored the views of primary care providers, in the context of cardiovascular risk prediction [4]. Lack of consistency with a holistic approach to patient care, questions around model validity, concerns about increased workload and whether such models add value to the clinical assessment were raised as concerns by the clinicians interviewed [4].

To provide further insight into clinician and patient perspectives on prediction models, this article presents new research as part of a programme of work aiming to develop a prognostic model for predicting relapse of depression in a primary care setting [5]. This qualitative work was undertaken alongside a prognostic model development study, to guide development and future implementation [6]. We explored perspectives on prediction models generally and with a specific focus on risk prediction in a primary care mental health context (relapse of depression in primary care).

The aims of the study were to:

- Understand general practitioners (GPs') current practice with respect to risk prediction models, including the ways in which they are currently used and communicated to patients, and
- Explore the views of GPs and people with lived experience of depression regarding communication of risk and the role of prediction models for relapse of depression in a primary care mental health context.

## 2 Methods

### 2.1 Qualitative Approach and Reflexivity

This was a qualitative study, grounded in a critical realist ontology (which holds that there is a reality to understand but that researchers can only ever partially know it) and contextualist epistemology (data do not directly represent reality, but reality is made sense of by the researcher's interpretative practices) [7, 8]. The study is reported according to the Standards for Reporting Qualitative Research [9]. Reflexivity is the process of critically reflecting on the process of knowledge production [10] and was a key consideration in our study. Reflexivity was explored and incorporated into the methods through the use of a reflexive journal and regular reflective discussions with the whole research team (Supporting Information S1: Material 1).

### 2.2 Public and Patient Involvement and Engagement (PPIE)

PPIE has underpinned this work from its conception. A Patient Advisory Group (PAG) contributed to the research questions and funding application; commented on drafts of materials for ethics application; co‐designed patient information materials; contributed to pilot interviews; co‐developed the semi‐structured topic guide; and contributed to the interpretation of findings (Supporting Information S1: Material 2). Hughes and Duffy outlined the different operational definitions of PPIE [11]. We used a combination of embedded consultation (members of the public are consulted regularly throughout the research cycle, giving feedback on research ideas and dissemination plans) and collaboration/co‐production (members of the public contribute to key decisions and findings as part of a steering group) throughout. We ensured a commitment to clear and agreed meaning and purpose of involvement through regular meetings and correspondence.

### 2.3 Recruitment and Sampling Strategy

We recruited adults (aged 18 years old and older) with lived experience of depression (excluding those with a history of severe mental illness) and GPs working in the English National Health Service (NHS; partner, salaried or locum GPs). We used purposive sampling [12], aiming for maximum variation in factors that we determined were likely to be most important in impacting on participants' experiences. For people with lived experience of depression, these factors were age, gender, ethnicity and index of multiple deprivation (IMD) of home postcode. For GPs, these factors were number of years working as a GP, contractual status, ethnicity and IMD (practice level). The NIHR Clinical Research Network (CRN) Yorkshire and Humber supported with recruitment through 15 general practices. People with lived experience of depression were recruited through a database search of GP electronic health record (followed by a letter of invitation to participate) or study advertisement through participating practices. GPs were recruited through a combination of study information being shared through participating practices, social media advertisement (Twitter/X) and snowball sampling [12, 13]. Potential participants were asked to express interest in participating by directly contacting the research team.

### 2.4 Data Collection and Analysis

GPs and people with lived experience of depression were interviewed concurrently between May and October 2022 by a single researcher (A.S.M.). Semi‐structured interviews were guided by topic guides (see Supporting Information S1: Material 3), and these were modified iteratively as further data were collected and analysed. Interviews were conducted online and digitally recorded using Microsoft Teams. Transcription was done by an external professional audio‐transcription company in the naturalised ‘intelligent’ verbatim; anonymised transcripts were stored on a secured University drive.

Data were analysed using NVivo 13 [14]. We used a form of codebook thematic analysis (TA), in which a coding framework is developed inductively during analysis to guide the generation of themes and researcher subjectivity is recognised as a resource for research rather than a limitation to be controlled for [15, 16]. Throughout analysis, we incorporated principles of constant comparison, whereby new data were systematically compared with previously analysed data, looking for similarities and differences [17]. Analysis was carried out by a single researcher (A.S.M.); to ensure consistency, a random selection of transcripts was coded by two researchers (A.S.M. and C.A.C.‐G.), and the coding was regularly reviewed by the whole multidisciplinary team and PAG. Sufficient sample size was determined by principles of data saturation [18] and information power [19]. Through constant comparison [17], we were able to identify the point at which no new themes (new ideas and meanings) were apparent upon collection of further data. We reviewed the richness of the information in the data set and the extent to which this met the aims and objectives of the study at regular multidisciplinary research team meetings. Trustworthiness [20] of analysis was enhanced through triangulation of findings within the multidisciplinary team and PAG; ensuring an adequate amount of rich data; comparisons and contrasts between data from two participant groups (GPs and people with lived experience of depression); and transparency in reporting data collection and analysis methods [21].

### 2.5 Ethical Considerations

Ethical approval for this study was granted by the NHS's Health Research Authority on 11 March 2022 (REC reference22/WM/0022). All participants provided written consent to participate and a risk protocol was available in the event that a participant disclosed suicidal ideation or intent. All participants were reimbursed for their time in the form of vouchers.

## 3 Results

### 3.1 Summary of Participants

Twenty‐three people with lived experience of depression and 22 GPs were interviewed (Table 1).

**Table 1:** Summary of participants.

People with lived experience of depression (*n* = 23)		
Gender	Male	*n* = 12
	Female	*n* = 11
Mean age in years (range)	45.7 (24−75)	
Ethnicity	White	*n* = 21
	Asian British	*n* = 2
Socioeconomic status (IMD^a^ decile, individual)	1−5	*n* = 10
	6−10	*n* = 13
Mean interview length in minutes (range)	55.9 (34−80)	
General practitioners (*n* = 22)		
Gender	Male	*n* = 10
	Female	*n* = 12
Ethnicity	White	*n* = 15
	Asian	*n* = 6
	Black African	*n* = 1
Job role	Partner	*n* = 11
	Salaried GP	*n* = 10
	Locum	*n* = 1
Socioeconomic status (IMD^a^ decile, practice^b^)	1−5	*n* = 6
	6−10	*n* = 15
Mean years of experience as a GP (range)	10.4 (1−30)	
Mean interview length in minutes (range)	53.3 (39−76)	

^a^ English indices of deprivation 2015 Lower Layer Super Output Area (LSOA) http://imd-by-postcode.opendatacommunities.org/ Decile (1 = most deprived, 10 = least deprived); https://fingertips.phe.org.uk/profile/general-practice/data

^b^ One GP participant ‘N/A’ as locum.

Illustrative data examples are presented to support the findings from the analysis. Participants are identified by a label indicating type of participant (P for person with lived experience or GP; gender [M or F]; and age).

### 3.2 Use of Prediction Models in Primary Care: The GP Perspective

This section presents findings from the analysis of interviews with GPs only, because this theme is about how GPs use prediction models and therefore was not relevant to people with lived experience. GPs reported using prediction models in primary care for the purposes of both diagnosis and prognosis. The findings are presented in terms of which models are used; why some models are used over others; and how prediction models are used in general practice.

#### 3.2.1 Which Prediction Models Are Used in General Practice

A relatively small number of prediction models were reported to be used (Table 2), all for physical, rather than mental, health presentations.

**Table 2:** Table outlining prediction models reported as being used in primary care.

Name of model	Purpose of model
Prognostic prediction models	
QRISK [22]	Predicts 10‐year risk of developing cardiovascular disease
CHA_2_DS_2_‐Vasc [23]	Prediction of stroke risk in people with atrial fibrillation
HAS‐BLED [24]	Predicts major bleeding risk in people with atrial fibrillation who are anticoagulated
FRAX [25]	WHO fracture risk algorithm for people with osteoporosis
ORBIT [26]	Predicts major bleeding risk in people with atrial fibrillation who are anticoagulated
Diagnostic prediction models	
NAFLD fibrosis score [27]	Predicts the presence of liver fibrosis in people with nonalcoholic fatty liver disease (NAFLD)
FIB‐4 [28]	Also predicts the presence of liver fibrosis in people with NAFLD
QCancer [29]	Predicts the risk of having cancer
Well's scores [30]	Predicts the presence of deep vein thrombosis and pulmonary embolism
FeverPAIN [31]	Predicts the presence of bacterial (Streptococcal) pharyngitis in people with sore throat

#### 3.2.2 Why Prediction Models Are Used

Two key themes were generated: some models are perceived to be useful (i.e., GPs are internally motivated to use them) and some models have to be used (GPs are externally motivated to use them, e.g., to meet financial or contractual targets). These two attributes are not mutually exclusive, but if neither applies, then this study suggests that GPs are unlikely to use the model in practice. The analysis also showed some additional key facilitators and barriers to models being routinely used in practice.

##### 3.2.2.1 Some Models Are Perceived to be Useful

Models were perceived to be useful when they guide discussions with patients, particularly when the GP perceives that the patient may not be satisfied with their suggested treatment options. An example of this is using a diagnostic prediction model to provide patients with an objective measure of the likelihood of viral infection rather than bacterial infection, to justify a clinical decision that could lead to difficult discussions with patients:

The FeverPAIN score is really helpful. I don't use that, necessarily, for my own clinical judgement but I use it for my patients, because I think when I'm not prescribing antibiotics it can be a difficult conversation. But actually, when I say ‘there's a really good and clever reason and I can reassure you for that’, then I think patients take it better than saying ‘I'm a really clever doctor and I don't think you need antibiotics’.

GP6‐M‐33

All GPs reported being more likely to use prediction models when they are perceived to directly influence clinical management. This was particularly the case when they perceived that the model adds something above what their clinical assessment (history and examination) can offer:

I think also if I can see a need for it…For the QRISK, deciding on whether the patient should have a statin, I need that evidence. Because a lot of the times, patient will have a high cholesterol but actually their risk can be one per cent. And I would have started them on a statin unnecessarily and vice versa.

GP2‐F‐38

GPs generally perceived a model as useful if it is known to be trustworthy and valid. Most GPs were interested in the evidence base for prognostic models, particularly those they used commonly, but said that they usually would not consult or critically appraise the primary evidence themselves. Usually, the fact that prognostic models were endorsed by the NHS or the National Institute for Health and Care Excellence (NICE) assured GPs that the models were sufficiently supported by evidence to warrant using.

If they were on NICE guidelines that gives me a lot of confidence that they are rigorous, as they've been well researched…For things that I'm using all the time, like QRisk, I like to have a better understanding of the population that it was studied…So, things that are more relevant to my population, I think, it's good to be able to explain that to patients.

GP1‐F‐35

Use of models by specialist colleagues, publicity in the medical press and incorporation into clinical templates also increased GPs' confidence in using tools and gave GPs the impression that they are widely used and accepted.

I suppose ones that you see specialists using…you'd feel a bit more confident because if your local consultants are using it, then you'd probably feel like it's a reasonably validated tool.

GP10‐F‐44

Most GPs reported being more likely to use models earlier in their career, particularly if they had been a feature of their GP training or formal medical education. All GPs also reported perceiving models as useful if they can be proven to save clinician time overall. Finally, all GPs reported valuing prediction models that they perceive to be potentially medicolegally protective:

In some situations, you're not safe if you don't use it. So, you know, if you're determining a few things, if somebody has DVT you, sort of, have to demonstrate that you made a safe decision by using a Wells score… from a point of view of documentation and medicolegal aspects.

GP18‐F‐34

In summary, GPs perceived prediction models to be useful for four key reasons: they guide discussions around management with patients; they aid clinical decision‐making; they are perceived as valid (trustworthy); or they protect the GP (e.g., medicolegally).

##### 3.2.2.2 Some Models Have to be Used

GPs reported the external motivators that make them think that a model has to be used. These included financial incentivisation (for example, through Quality Outcome Framework [QOF]), Care Quality Commission (CQC) recommendations or inclusion in practice policies and local referral pathways:

If it [prediction model] was really evidenced, cost‐effective and worked for patients and QOF got a hold of it and told us that there was a financial incentive to do it, I think that would probably encourage practices to put more emphasis on encouraging their GPs to use it.

GP3‐F‐32

Being adopted as part of a practice or local policy was another thing that made GPs more likely to use a model.

Sometimes it's a practice protocol and policy, sometimes it's QOF‐related, it's just your local sort of protocols and guidelines and things as to what you follow as well as just good practice. So I think it's just a mixture really of things.

GP8‐F‐52

##### 3.2.2.3 The Time‐Limited Consultation

**Figure 1:**
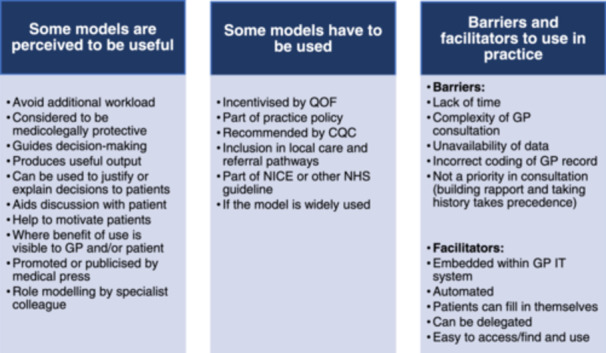
Summary of GP perspectives on prediction models in general practice.

GPs discussed the practical barriers and facilitators to using models in practice (Figure 1). Limited time and resources were viewed as major barriers, with prediction models being one of many competing considerations in a consultation. Other barriers were limited reminders to use the particular tool and limited accessibility of the model (i.e., not integrated into practice systems). All GPs discussed the increasing complexity of the GP consultation generally, with many different demands on that relatively short amount of time and multiple different issues requiring discussion.

I think a lot of this is down to time, isn't it, most appointments are still ten minutes…when you manage someone with mental health issues, ten minutes, even 15 minutes isn't enough at all, and then to then open up a pop‐up with lots of questions, it's going to take another five, ten minutes to do, so it's just it's time constraints more than anything else.

GP11‐M‐40

Given the pressures outlined, most GPs suggested that the outputs and recommendations from models had to be realistic within the clinical context. Where demands arising as a result of models were overly onerous or unrealistic, most GPs did not feel that it was an efficient use of their time to use a model. Other facilitators to use were integration with the electronic clinical record; models that can easily be delegated to other non‐GP members of the primary care team (e.g., practice nurses) or filled in by patients directly; and models that do not require additional work to utilise.

So it would need to be like a template that's accessible that people can find on their computer system, via Ardens or whatever, and then kind of recorded into the computer system, that's always preferable. It'd need to be really quite simple because some people are more IT‐literate than others.

GP15‐F‐47

#### 3.2.3 How Prediction Models Are Used

GPs discussed the practicalities of using prediction models in general practice, including dealing with missing predictor information and borderline outcomes. Most GPs reported that where information was missing, they would endeavour to collect the necessary information to enable predictions to be as accurate as possible, as long as it was felt safe to wait for additional information.

I always try and get up‐to‐date information. So, you know, the classic thing is we haven't got their blood pressure or we haven't got their weight or their cholesterol's out of date. And I think if we're going to do it, we should do it properly really.

GP2‐F‐38

Borderline results were used by most GPs to initiate discussions with patients around shared management decisions, for example, in the context of using QRisk:

Yeah, so for me, if someone's borderline, I take into account what the patient thinks, and if they have strong feelings either way about starting medication. If you've got someone very motivated to make lifestyle changes, I would tend to let them try that, and then try and arrange a follow up appointment to review how that's going. If they don't feel that they can make any lifestyle changes…then I'd probably err towards prescribing.

GP14‐F‐45

In summary, prognostic models in general practice are usually used in an advisory and pragmatic way to facilitate individualised shared decision‐making with patients.

### 3.3 How to Communicate Results of Clinical Prediction Models

The remainder of this article reports findings from analysis of data from both GPs and people with lived experience of depression (see Supporting Information S1: Material 4 for further comparative illustrative data extracts). Participants discussed the kinds of outputs from models that facilitate discussions, and preferences around statistics and visualisations. Most GPs and people with lived experience of depression did not think that numerical information around risk was appropriate or necessary for all patients. It was suggested that patient preferences around such information are explored and that the level of detail provided is tailored to the individual patient. Some people with lived experience of depression felt that broad risk categories are sufficient and felt that people were not keen on hearing numerical information to back these up.

I think low, medium, and high is more than sufficient…a lot of people glaze over when you start talking about you've got 63 per cent chance of this and that sort of thing, it doesn't really mean much…I think people have a tendency just to glaze over when they start hearing the numbers.

P7‐M‐48

Some GPs agreed that keeping the output simple and using risk categories would avoid the explanation and discussions with patients from becoming too confusing or complicated.

I think just something more simple, I think it's just like three categories really, low, medium, or high, and they understand that. We make it too many different levels, then it just gets a bit confusing everyone and they're not really sure what to make of it.

GP11‐M‐40

A variation on risk categories (e.g., high, medium and low risk) suggested by some GPs and people with lived experience of depression was a traffic light system or red, amber, green (RAG) rating. People with lived experience of depression thought that RAG ratings were acceptable, although they did raise the potential issue of using ‘red’ ratings for people who are anxious and felt that this might need some additional consideration.

You could have like a RAG rating…but again, it's whether you want to create the connotation of ‘you are red risk’, which is probably not great for someone who's quite anxious.

P17‐F‐36

Most GPs suggested that broad risk categories are vague and it is important to quantify risk if asked to by patients.

I think it depends on the patient, doesn't it, some people would want to know what high was, so what does that convert to, what percentage is it, so some people would want that translating into some hard data that they can then understand, whereas other people, low, medium and high is probably all the information that they want to know, and that might be good enough for them to make a decision accordingly.

GP10‐F‐44

Proportions were generally viewed as preferable to percentages because they were seen as easier to understand and also more personal. One of the main reasons both groups of participants thought this was the case was that using proportions enabled people to see themselves as part of the explanation.

From a layperson's point of view, I would probably think proportions would work better, they can see themselves one in ten, so that's one person in ten, that's them, whilst ten per cent is the same, but that's just a bit more wordy, a bit more numbery, they may not fully understand it.

GP11‐M‐40

Most people with lived experience of depression agreed with this and felt that this made the explanation seem to be more tailored to the individual, reassuring and easier to visualise.

I think if you put it like you just said in a group of people, yeah, that would be better, for me anyway…in 40 per cent median of people you'd think you're not on your own, that there's another 39 per cent stood behind you. There's 39 stood there with you.

P6‐F‐52

Finally, visual representations of risk were viewed as useful by all participants:

I quite like the visual representations which are reflected in percentages…100 green faces, three of them are sad, two of them are dead, those kind of things I think are much easier to demonstrate risk than just talking through numbers.

GP17‐M‐35

It's obvious that people have a different way of learning… I must admit, I can visualise things better by actually seeing them, I mean percentages and things but sometimes, yeah, an actual diagram…that would make it a lot easier and simple for people to understand.

P13‐F‐58

In summary, GPs and people with lived experience of depression reported that numerical information can be useful but ideally, explanations involving numbers should be tailored to individual patients' preferences. Visual representations, such as Cates plots (which use smiley faces to visually represent risks and benefits of a particular treatment), can be helpful for explaining risk to patients.

### 3.4 Prognostic Models for Depression Recurrence in Primary Care

Here, we report the perspectives of GPs and people with lived experience of depression with respect to risk prediction for relapse and recurrence of depression in primary care. There was a view from some with lived experience that depression (and mental health conditions generally) is more personal and individual than physical health conditions and therefore potentially more difficult to apply statistical models to.

Mental health is such a personal thing, it's something that's so hard to put a statistic on or do averages and things on because everything's so personal. I don't know, I don't think it's even scientifically possible to put an exact number on anything to do with anything like this, so I don't think it's even worth trying.

P10‐M‐29

In contrast, others with lived experience of depression suggested that the comparison with physical health problems could be helpful and thought that applying similar models to those that already exist for physical health conditions might be comforting or reassuring to people with depression.

It almost makes it more digestible, doesn't it? It makes it more similar to physical health conditions that people are more familiar with. Like quantifiable – almost like you can measure depression, you know? Like you can measure blood pressure or something like that. Like that might provide a source of comfort for people, rather than it being this intangible thing that's going on in your brain.

P16‐F‐24

Most GPs had similar concerns around trying to predict outcomes in depression, compared with physical health. It was felt that there would need to be a possibility for a greater degree of individualisation with prognostic models for mental health than for physical health.

I think depression is one of those things that everybody is affected by it so differently, that I just think that we always have to be careful with mental health tools that there is an element of individualisation and personalisation available. I think it's difficult with risk stratification tools, isn't it, because you're trying to put in data and have an answer. Mental health is always a bit more grey than black and white, than some other medical problems, isn't it.

GP21‐F‐33

Participants discussed the specific topic of prognostic models for relapse risk prediction, whether there is a role for a relapse risk prediction model and how this might look in practice. Overall, most GPs and people with lived experience thought that a relapse risk prediction model would be useful, so long as there were options to help those people who were identified as high risk. GPs discussed the feasibility of incorporating such a tool into a consultation and, in line with the earlier discussion around barriers, felt that as long as it was focussed and not overly long, it would be acceptable to GPs.

You've got to be able to fit it into a ten‐minute consultation, but at the same time, if it's going to save the patient a relapse and more time in the long‐run, then I guess that's the thing to be thinking about. So, I think it would be fine as long as it wasn't too onerous and you could actually get through it.

GP8‐F‐52

People with lived experience of depression discussed the importance of having a plan in response to the risk prediction and that it would not be helpful to be told one is high risk without additional support being provided. Most felt that this need not necessarily be as substantial as a relapse prevention intervention but should at least include some relapse prevention planning and discussion of early warning signs of relapse.

To me, that would all be part of somebody saying, you know, we are looking at this holistically. We're not just, you know, bandaging you up and getting rid of you. We care about what might happen downstream…Where I would find it gets a little bit difficult is if somebody said you are high risk of relapse but then there were no actions in place to mitigate that.

P8‐M‐57

GPs discussed how a risk prediction model might help with using resources more effectively and enable GPs to more effectively identify patients for whom different follow‐up strategies are more appropriate.

I guess the advantage would be that you would have something that you could then justify saying, this person needs a regular appointment, say every three to six months to just check in on them and how they're doing and make sure that things aren't going south for them.

GP21‐F‐33

GPs also highlighted further implications of such a tool, including patients' access to medical records and medicolegal considerations.

My worry is, that because patients have access to their records any score like that, that's written in your records, has the potential to frighten a patient, if they read it without being in a consultation or having it explained to them. So I'd want it to be something that was discussed with patients, rather than just automatically added to the patient's records, because I think that could be quite harmful.

GP14‐F‐45

The medicolegal aspects referenced were raised in a way that was supportive of a risk prediction tool, but also raised caution about some of the implications. First, risk stratification could be used to justify decisions and give GPs some evidence‐based medicolegal justification for clinical decisions taken:

I don't want to keep coming back to medicolegal aspects because our job is so much more than that, but if one of my patients has a stroke on an anticoagulant and I have to attend coroners' court to explain their treatments, it is very, very easy because I've got the data, I've got the evidence base behind it. If the same was in mental health, it would be lovely if we could say we had done this tool, this patient posed as moderate risk, so this was introduced, it would be very useful.

GP17‐M‐35

However, calculating individualised risk of relapse and failing to offer an appropriate plan in response were felt to have the potential to leave GPs feeling medicolegally vulnerable, which reinforces the need for any model developed to be implemented with advice around actions to be taken.

Unfortunately, with this increasingly litigious society, my mind goes to, well, if I've got a relapse tool and either I've chosen not to use it or even worse, I've used it, recognised a patient is at high risk of relapse, the patient then relapses and I'm seen to have recognised that but then done nothing, you wonder, well, goodness me, am I going to be in trouble… So yeah, I think it could be useful, but there'd have to be some sort of caution with it I suspect.

GP17‐M‐35

## 4 Discussion

### 4.1 Summary of Key Findings

GPs reported using prediction models if they perceive them to be valuable and/or that they have to use them as part of their contractual obligations. If neither of these criteria are met, prognostic models are unlikely to be used in general practice. If these criteria are met, then there are still practical barriers to use (e.g., time constraints in a consultation) that must be overcome and facilitators to use (e.g., integration with the GP IT system) that should be considered in any implementation process.

People with lived experience of depression reflected that they were keen to learn about their outcome risks and there is likely to be value in producing numerical and other kinds of output (e.g., risk categories) and allowing the patient and GP together to decide on the most appropriate information to guide a discussion tailored to the individual patient. There was more scepticism from participants about the use of prognostic models for depression relapse compared with physical health conditions, due to the perceived more ‘subjective’ nature of depression. However, some people with lived experience of depression reported that they would find an approach more in line with physical health to be reassuring. The importance of an appropriate action plan or additional support for higher risk individuals was highlighted by all participants.

### 4.2 Comparison With Previous Literature

There have been a limited number of previous studies exploring clinician views of prediction models and only one in primary care [4]. One study explored the views of mental health clinicians and healthcare administrators on prognostic models in the context of suicide risk prediction [32]. In that study, clinicians shared the concerns of participants in our study that they would not have the clinical resources to adequately meet the potentially increased demand, as well as having medicolegal concerns. Like in our study, participants thought that, once a clinician is aware of risk information, they feel that they would be personally liable for not acting quickly enough. Currently, there is limited guidance for helping clinicians understand and mitigate the risk inherent in using risk prediction models to guide medical treatment [33].

Our results are further supported by preexisting literature on the value of models that help facilitate discussion with patients around prognosis and guide patient management [34, 35]. Only one preexisting study explored patient perspectives of prognostic models, in the context of communicating prognosis to people with head and neck cancer [36], in which patients were broadly accepting of the use of prognostic models and keen to understand the quantitative results. Our findings suggesting that integration with clinical systems is a facilitator to use are supported by a previous study looking to incorporate the FRAX tool into a nurse‐delivered care review in general practice [37].

A recent scoping review looked at the evidence around communication of prognostic model results by healthcare providers to patients [38]. The review reported a lack of evidence on the best ways to communicate prognostic model results. It did, however, highlight the importance of contextualising information when discussing with patients, which is consistent with our findings. This contextualisation specifically referred to defining timeframes over which predictions are applicable, using results in the context of shared decision making and using data visualisation as an explanatory tool (particularly for those with lower health literacy).

The findings from our study suggest that communication of risk benefits from being individualised to each patient in question. This is supported by previous research on the subject of risk communication that has found that either percentages, frequencies or proportions are acceptable for conveying numerical risk to patients; the use of visual representations in multiple formats helps cater for different patient preferences (illuminated with words and numbers where possible); and the importance of initially assuming low numeracy and providing optional additional detail as desired (rather than providing more complex information to start) [39, 40, 41]. An additional consideration for further studies of clinician and patient perspectives around clinical prediction models is a better understanding of the communication of uncertainty around risk estimates. There has been some previous research exploring this in the context of prognostic models in cancer, which found heterogeneity in the ways in which uncertainty is both available to and understood by clinicians and communicated with patients [42]. The extent to which there is uncertainty around estimates is of significance to researchers and often measured and reported in the form of 95% confidence intervals. Whether confidence intervals would be useful and easily understood by clinicians and patients in the context of individual risk estimates from prediction models is unclear and warrants further qualitative exploration.

### 4.3 Strengths and Limitations of the Study

Participants were recruited from populations with a broad range of demographics in both groups. The sample of people with lived experience of depression had fewer people from ethnic minority backgrounds than we aimed for, despite efforts to increase this by purposively approaching practices with higher ethnic diversity in their patient populations. Thus, the perspectives of people from different cultural backgrounds may not be well represented here. Future work should further consider the views of people with lived experience of depression from a wider range of ethnic minority backgrounds. Apart from a random subsample of five transcripts that were coded by two researchers (A.S.M. and C.A.C.‐G.), the rest were coded by a single researcher (A.S.M.). This is consistent with the theoretical approach and form of codebook TA used, but is worth taking into consideration when interpreting our findings.

A key strength of this study was the involvement of the PAG, which was involved from study conception. This group contributed to the design of patient information materials and the development of the semi‐structured topic guide, to ensure that the topics discussed were relevant and important to patients. They further contributed to the interpretation of findings, adding new insight to the results of these qualitative analyses.

### 4.4 Implications for Practice and Research

Prediction model researchers should be cognizant of the clinical context, before starting model development. Predictions made in primary care must relate to predictors and outcomes that are important to GPs and patients. Our study suggests that, if GPs do not view the model as useful and are not otherwise externally motivated to use it, the model will not be used in primary care. Researchers are advised to consider developing models that provide a range of outputs (both numerical and visual) to allow discussions between patients and clinicians to be tailored to individual preferences.

Primary care mental health is an area where prediction models may be useful. Models have been developed to predict outcomes in psychosis [43], depression onset [44] and depression outcomes [45], and to guide depression management [46] in primary care with promising predictive performance. This study has demonstrated that predicting relapse and recurrence of depression in primary care would likely have useful applications, although models have demonstrated limited predictive performance thus far [47]. As in previous studies [32], participants in our study highlighted the importance of having an action plan or ability to provide additional support as appropriate, in response to the findings of prediction models. Previous literature suggests that appropriate approaches for people with increased depression relapse risk might include case management and relapse prevention planning [48, 49], as well as more formal relapse prevention interventions [50]. Researchers aiming to develop clinical prediction models in the area of primary care mental health should be aware of potential barriers to model use, including perceived increased complexity and challenges in predicting outcomes when compared with prediction models for physical health conditions. When developing prediction models in this area, we recommend embedded and meaningful public and patient involvement and concurrent qualitative research, ideally as part of a mixed‐methods approach.

## Author Contributions

**Andrew S. Moriarty:** conceptualisation, analysis, funding acquisition, investigation and administration, methodology and writing–original draft preparation. **Joanne Castleton:** conceptualisation, funding acquisition and writing–review and editing. **Dean McMillan:** conceptualisation, funding acquisition and writing–review and editing. **Richard D. Riley:** conceptualisation, funding acquisition and writing–review and editing. **Kym I. E. Snell:** writing–review and editing. **Lucinda Archer:** writing–review and editing. **Lewis W. Paton:** writing–review and editing. **Simon Gilbody:** conceptualisation, funding acquisition and writing–review and editing. **Carolyn A. Chew‐Graham:** conceptualisation, analysis, funding acquisition, methodology and writing–review and editing.

## Ethics Statement

Ethical approval for this study was granted by the NHS's Health Research Authority on 11 March 2022 (REC reference22/WM/0022).

## Conflicts of Interest

Carolyn A. Chew‐Graham is Editor‐in‐Chief of Health Expectations. The other authors declare no conflicts of interest.

## Supporting information

Supporting information.

## Data Availability

Data will be stored for 10 years and we would be happy to share anonymised data with other researchers on reasonable request.
