# Supplementary material for: The Value of Clinical Prediction Models in General Practice: A Qualitative Study Exploring the Perspectives of People With Lived Experience of Depression and General Practitioners
Source: Health Expect. 2024 Dec 18;27(6):e70059. doi: 10.1111/hex.70059 (PMC11655671; doi:10.1111/hex.70059)
Supplement: Supplementary file 1 — Supporting information. [file HEX-27-e70059-s001.pdf]

Supplementary Materials

Table of Contents

Supplementary Material 1 2

1.1. Reflexive statement and interviewer details 2

1.2. Overview of thematic analysis and roles of research team and patient advisory group 6

Supplementary Material 2: Public and patient involvement and engagement 7

2.1. Details of patient advisory group 7

2.2. Overview of PPIE activities throughout study 8

Supplementary Material 3: Semi-structured topic guide 9

3.1. Topic Guide for people with lived experience of depression 9

3.2. Topic Guide for GPs 12

Supplementary Material 4: Table summarising data from both groups of participants for each theme 15

Supplementary Material 1

1.1. Reflexive statement and interviewer details

Details around the lead researcher and interviewer are presented here to provide additional context for the reader:

The lead researcher and interviewer (ASM) is a male GP partner and a researcher (an NIHR Doctoral Research Fellow at the time of analysis, now an NIHR Clinical Lecturer in Primary Care). His professional qualifications are presented on the title page of this paper. He knew three of the GPs interviewed in a professional context only (through professional networks). The rest of the interviewees were not known to him prior to the interviews.

The following reflexive statement is intended to give further context for the reader, to be read alongside the findings:

In terms of functional reflexivity, all interviews were done remotely due to restrictions imposed by the COVID-19 pandemic. This was enabled by the increased familiarity and computer literacy of people as a result of the pandemic, in particular a willingness to use Microsoft Teams. This opened up a much wider participant pool and worked well from a flexibility point of view. However, Braun and Clarke (2013) discuss drawbacks of remote interviews, which can sometimes mean that non-verbal communication is less well conveyed and can affect data generation and interpretation. The use of virtual interviews may have limited the amount of information people were willing to share. There were only technical problems with two of the interviews and these were minor (slight screen freezes or parts where the audio froze or cut out). There were also occasional interruptions (doorbell ringing, participant received a phone call) and some of the GPs were in their consulting rooms which created the impression that they had limited time or even that they could be interrupted for a clinical reason, which changed the dynamic very slightly. I do not think these affected the data collection and caused only minor disruptions overall. The selection of semi-structured interviews as my data collection tool meant that the conversation progressed according to my agenda rather than the participants, although there was plenty of time for me to allow participants to talk and explore tangential avenues of conversation. There was only one interview where I found it very difficult to keep on track due to the patient being extremely talkative and wishing to discuss her life more generally and secondary care presentations more than the subjects I was intending to explore. This interview certainly yielded useful (and, actually, fascinatingly contradictory data) but was the only interview where I felt I had to explicitly ask if we could focus more on the topics of my choosing. The participant was very understanding about this and I do not think this affected the rapport we had developed over the hour.

In terms of personal reflexivity, there are certain values and characteristics that I possess that situate me as a researcher. First, from a socio-demographic perspective, I am male, white and, of particular relevance, I am a GP and therefore viewed the data from the perspective of one group of participants (other GPs, with whom I was an "insider" researcher), but not the other (people with lived experience of depression, where I was more of an "outsider"). Furthermore, I brought a research background to this qualitative project that was more quantitative in nature – the majority of my previous research projects have been carried out within a scientific, positivist (or at least post-positivist) paradigm. My natural inclination is to think in terms such as bias, generalisability and reliability and orienting myself to a qualitative methodology was not an immediate process but one that took some introspection, discussion with my supervisors and lots of reflection and reading. Finally, many of my mannerisms and approach to interviewing were inevitably influenced by my clinical training, and particularly the training I have had in taking a medical history. A commonly used framework in medical training, and one that I was trained using, is the Calgary-Cambridge framework, where open questions are used initially and become gradually more focussed as the consultation progresses. I undertook qualitative research training as part of my Fellowship to ensure that I was not leaning too heavily on my clinical interview training and was adopting a suitable approach for qualitative research.

I also hold particular political views with respect to the governing of the country generally but, more relevantly, regarding the funding and resourcing of primary care. I am regularly engaged in political discussions and aware of medico-political advancements and changes. In particular, I am aware of how pressured and under-resourced primary care is at the current time. Some of the GPs I interviewed shared similar views as me, and my knowledge in these areas led to interesting conversations about primary care mental health provision and the implementation of relapse prevention in primary care. However, my view as a GP was not compatible with some of the expectations expressed by people with lived experience and I had to be more vigilant of my biases where discussions around GP resource were raised; my purpose in these interviews was to learn about patients' experiences and views and not to challenge these unduly.

I felt it was important to explain to all participants at the outset that I am a GP. I tried to remain cognizant of the ways in which this might have affected people with lived experience of depression. For example, I am aware of the potential power dynamics that could be perceived and felt by people with lived experience of depression. There is also a reverse power dynamic in that people with depression may view me as someone who represents a profession who is supposed to serve and help them and that there may be some criticisms of the care that they have received from their own GP. My clinical background also meant that there was a risk that my interpretation was overly clinical and less related to the meaning in the data.

Discussing my clinical role as a GP was particularly interesting in the case of the GP interviews. Some of the answers given by the participants gave the impression that they felt as though they were being tested: for example: "I don't because I…I might be wrong here, you can instruct me afterwards, but…" [GP9]. This fits with previous evidence on the subject (1) of GP researchers interviewing GPs. In keeping with this previous literature on the subject, my sense was that being a GP interviewing GPs had some benefits in terms of building rapport and generating rich data. Chew-Graham described the role of a GP researcher interviewing GPs as that of a "professional peer and private confidante" (1). There was a sense in which some of the conversations could begin at a point of exploring the important questions rather than too much explanatory dialogue or context-setting (on either part). There was also a feeling that the interview participants understood that I had some pre-existing insight into some of the clinical issues and, in some case, I think the interviewees opened up more than they might have done if I had been a non-clinical researcher.

Where this had perhaps a less positive effect was in cases, particularly in earlier interviews, where I realised on listening back to the interviews or reviewing the transcripts with my supervisor that I had colluded with the participant rather than remaining objective and exploring topics in more detail. In the later interviews, I made an effort to remind myself to step back from this and not to take the participants' statements for granted. I asked them to clarify where there was any ambiguity, even when their expectation appeared to be that I would know what they meant. As Chew-Graham (2002) reflects, the assumption of a shared understanding between the interviewer and interviewee in qualitative research is problematic. The aim of this study has been to understand the ways that GPs manage patients and try to make sense of clinical decision making. It is difficult to explore the more nuanced and less explicit factors influencing this if much of it is assumed to be obvious. There were also some instances where the GPs being interviewed asked me questions, as though I were an expert in the clinical area. There were others where GPs may have felt judged, or as though they were revealing something about their practice that they felt I would not approved of: for example, by prefacing answers with "I'll be honest…"

The qualitative work has really enabled me to contextualise the quantitative aspects of the project and helped me understand what they mean and what the implications are moving forwards (2). Because my previous research exposure has largely been quantitative, it has been a really interesting experience and learning point for me to come to understand that the interviewer can be actively involved in generating data and constructing meaning from the interviews rather than passively "collecting" it. Reassuring interviewees about confidentiality and adopting a non-judgemental approach was essential in ensuring that the data generated were as honest as possible, although the data must still be interpreted on the basis that some interviewees may have been holding some things back or even, potentially,

fabricating answers or modifying their responses to give what they felt was the "right answer".

I found it difficult in the first few interviewers to remain dispassionate and was worried about leading the interview participant or sharing my own thoughts. I found as the interviews went on that I became more comfortable and confident in sharing my own views, where appropriate, and sometimes engaging in a peer-to-peer conversation rather than an interview. I found that this helped to build rapport and engage the participant more. While I found it challenging when lay participants criticised or expressed negative views of GPs, I think it was important to recognise and accept that some patients have had less than satisfactory experiences with GPs and recognise that the aim of the research is hopefully to improve this.

1. Chew-Graham CA, May CR, Perry MS. Qualitative research and the problem of judgement: lessons from interviewing fellow professionals. Vol. 19, Family Practice. 2002.
2. Creswell JW, Klassen AC, Clark VLP, Smith KC. Best Practices for Mixed Methods Research in the Health Sciences [Internet]. 2011 [cited 2021 Jul 3]. Available from: http://obssr.od.nih.gov/mixed_methods_research/pdf/Best_Practices_for_Mixed_Methods_Research.pdf

1.2. Overview of thematic analysis[1] and roles of research team and patient advisory group

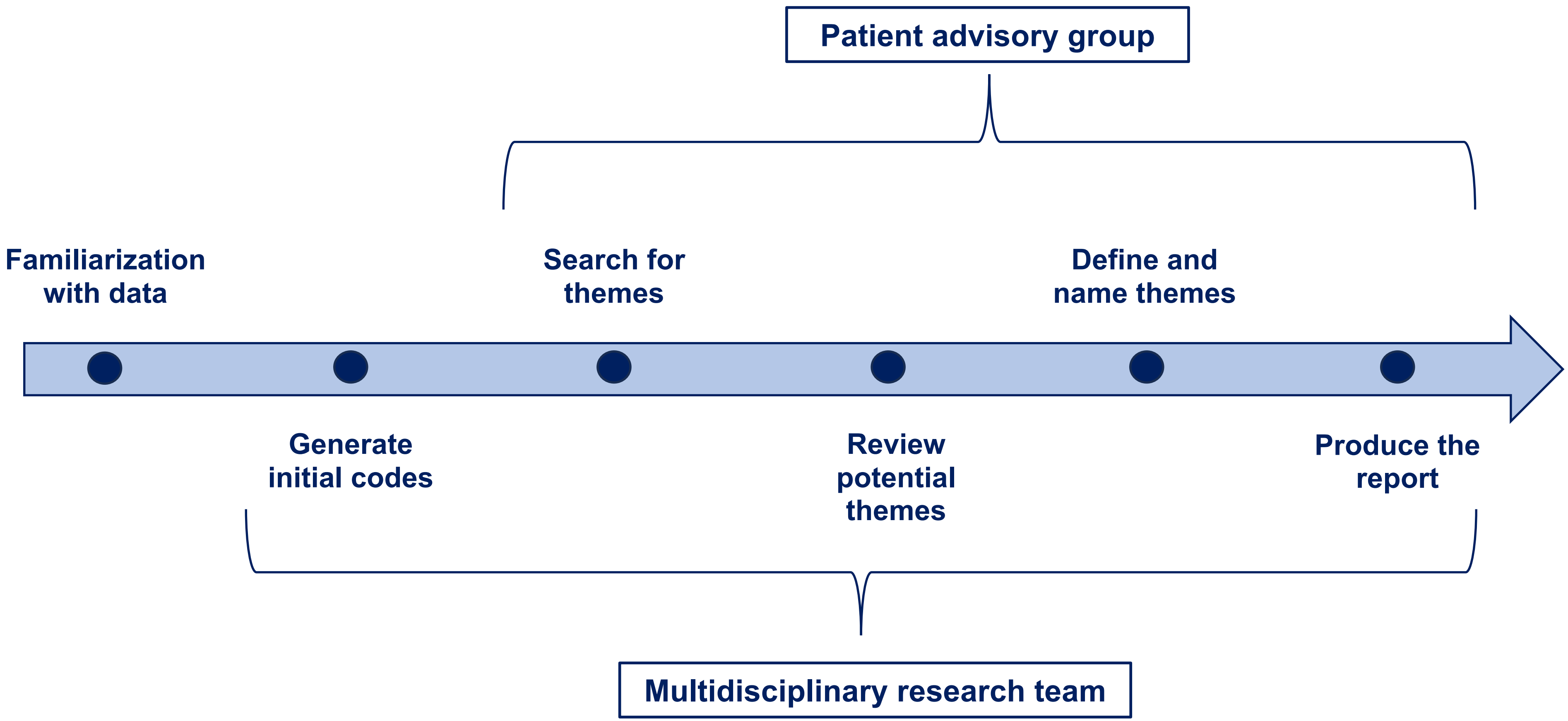

[1] Adapted from the six stages of thematic analysis (Braun and Clarke, 2006)

Supplementary Material 2: Public and patient involvement and engagement

2.1. Details of patient advisory group

Gender	Female	n=5
	Male	n=2
Age (years)	18-30	n=1
	30-40	n=0
	40-50	n=3
	50-60	n=2
	60+	n=1
Ethnicity	White	n=7
	Other	n=0

2.2. Overview of PPIE activities throughout study

Pre-PhD September 2017
- Overview of study proposal and group discussion
- Idea-sharing and prioritising research objectives
- PAG comment on draft NIHR application

October 2019
- Review and development of systematic review and quantitative protocols
- Preliminary discussions around qualitative workstream
- Discussion of training opportunities for PAG members

October 2020
- Review of Plain Language Summary for Cochrane Review by all PAG members
- Review and finalisation of prognostic model protocol
- Further preliminary planning for qualitative workstream

June 2021
- Workshop for co-production of participant-facing qualitative materials prior to HRA ethics submission
- Initial feedback from PAG on semi-structured topic guide

July 2021
- 2 x Pilot interviews with PAG members
- Feedback and revision of topic guide

September 2022
- Presentation of initial data analysis
- Discussion of preliminary themes and framework

April 2023
- Finalisation of thematic analysis
- Agreement on final themes
- Discussion of next steps including lay dissemination of findings

Supplementary Material 3: Semi-structured topic guide

3.1. Topic Guide for people with lived experience of depression

Introduction and consent

Introduction and consent

Demographics: age, post code, gender identity, ethnicity, level of education reached, (previous) employment, rural/urban/inner city, who is at home with you, other long-term conditions

GP details (in case risk protocol needs to be activated)

Please can you tell me why you decided to take part in this study?

1. Please can you tell me about your depression?

   Possible prompts:

   Are you currently experiencing depression?

   When do you think you had your first experience of depression?

   How many times have you experienced depression? How often?

   Do you have any thoughts on what is the cause/trigger for your depression?

2. Can you tell me who is/has been involved in helping you with your depression?

   Prompts:

   can you tell me what sorts of treatment you have had?

   How do you feel about your depression treatment, care and support?

   Is there anything you'd like to try or to have been offered - and what are the barriers to this?

3. Can I ask, were you aware or advised about the risk of having depression again after your previous experience(s) of depression?

Can you tell me about any discussions you had about this with your GP or anybody else?
Would this have been useful? Why/why not?

If you could be given more specific, individual information about your personal risk of becoming unwell again by your GP, how would you feel about that?

If available, how might this kind of information be delivered to you? Can you explain why?
e.g. by a healthcare professional, online (app, website)

In terms of having risk explained to you, what makes most sense to you? For example, some people talk in terms of percentage (60%), risk proportion (i.e. 6 in 10), or categories such as high, medium, or low risk.
What words should be used or not used? (e.g. high risk, significant risk)

[Read explanation of PREDICTR study]**

Are there any important things that you think are missing from this? Why do you think they are important?

4. Would you have found it helpful to be offered support to prevent relapse after your depression improved?

Prompts:

What kind of support you would prefer?

Examples: CBT, IPT, MBCT, medication

What sort of help do you think would help you decide the kind of support?

WOULD YOU LIKE THIS IN PRIMARY CARE??

5. Anything else you would like to add?

Close interview: Thank you, re-check consent, arrange reimbursement.

**(Example explanation of the PREDICTR tool): We are developing a tool to help GPs to assess a person's risk of relapse/recurrence after having depression. We have included the following information to help to make this assessment:

- whether a person has had depression before;
- whether they still have some symptoms of depression when they are feeling better;
- whether they have also experienced anxiety as well as depression;
- how severe their episode of depression was.

These were chosen after looking at the evidence and research literature. However, we understand that there may be factors that are important to people that we have not included.

3.2. Topic Guide for GPs

(Version 2.0, 19th May 2022)

Introduction and consent

Demographics: type of practice, urban/rural, training/teaching

AGE

GP – years of experience as a GP, Salaried/locum/Partner, ethnicity, gender

Does the GP have any other roles alongside clinical commitments

Does GP have any specialist interest/expertise in MH?
Why did you agree to be interviewed?

1. Can you reflect on your experiences of managing people with depression
    a. diagnosis
        i. Do you/would you routinely do PHQ-9/GAD-7? How do you use these instruments in practice?
    b. management options

    c. Monitoring (and explore their behaviour compared to colleagues in their practice)

    d. PT comment re unprompted follow up
    e. defining remission/recovery
    f. monitoring/review after remission/ recovery
    g. OTHER STAFF???
    h. What would help from a resource point of view??? Premises?? ARRS??
2. COVID

3. Can you tell me about if and how you assess risk of relapse in patients with depression?

4. Please can you tell me about a time that you had a discussion with a patient about relapse?

    a. What advice do you give? When?
    b. Do you advice a person about staying well and to self-monitor mood (eg using PHQ9)

5. Do you feel confident in being able to assess a patient's risk of relapse/recurrence once they have entered remission/recovery?

What sources of information or guidelines have you used/would you use to help you to assess this risk?
Do you think you can communicate risk to patients? Why? Why not?

6. What is your understanding of the type/availability of relapse prevention interventions for patients?
7. AWARE OF BOOSTER SESSIONS?? RELAPSE PREVENTION??
8. WHAT IS INTERFACE LIKE WITH IAPT / SECONDARY CARE???

is this primary care or need additional funding???

(Example explanation of the PREDICTR tool): We are developing a tool to help GPs to assess a patient's risk of relapse/recurrence after having depression. We have included the following information to help to make this assessment: previous depression (patient report/GP record); residual depressive symptoms (PHQ-9 at remission); whether they have also experience anxiety as well as depression (GAD-7); how severe their episode of depression was (PHQ-9 at baseline). These were chosen after looking at the evidence and research literature.

9. Are there any factors that you think should be included in this tool that we have not mentioned?

10. how you might use such a tool in practice? Do you think your colleagues would

What would be the best way of implementing this tool? (e.g. IT, paper, internet, patient self-complete)

Would you be prepared to use this tool if there was evidence for the effectiveness of such an approach?

What might be the barriers to using such a tool?

Would you have confidence in using this tool?

8. How do you think risk is best communicated to GPs and patients?

e.g. percentage risk, proportion, categories (high, medium, low risk)

Prognostic models:

- Do they want predicted risks, or a 'high' or 'low/normal' output
- If and how do they use and communicate predicted risks in practice
- How do they define risk thresholds for guiding treatment choices, and what if someone is just above or just below the threshold
- How do they implement models when some information (Eg smoking, BMI) is missing?
- Do they use computer, or a chart, or nomogram etc for implementation?
- Can patient's handle information from prediction models? How do they facilitate discussions of risk?
- What, if any, models do they use
- What makes them trust some models and not others

Is there anything you would like to ask or suggest?

Close interview: Thank you, re-check consent, arrange reimbursement.

Supplementary Material 4: Table summarising data from both groups of participants for each theme

	Participant group	
**Theme**	**General practitioners**	**People with lived experience of depression**
How to communicate results of clinical prediction models	*I think just something more simple, I think it's just like three categories really, low, medium, or high, and they understand that. We make it too many different levels, then it just gets a bit confusing and they're not really sure what to make of it.* GP11-M-40	*I think low, medium, and high is more than sufficient...a lot of people glaze over when you start talking about you've got 63 per cent chance of this and that sort of thing, it doesn't really mean much, I don't think. Like I say, I think saying somebody's got a high risk is better than saying you've got a 90 per cent risk… I think people have a tendency just to glaze over when they start hearing the numbers.* P7-M-48
	I was going to say traffic lights are always good…we need something really simple that we don't have to think about, so I think if you had like a traffic light, a red, amber, green, so green, low risk, don't have to do anything, red, need to do whatever, amber have a think about it, something like	*You could have like a RAG rating, bloody RAG ratings, there's a RAG rating for everything, but they are like, they do have their place. But again, it's whether you want to create the connotation of you are red risk, red risk ohhh, which is probably not great for someone who's quite anxious.*

	that…I think something really straightforward and simple would be better because we don't need anything else to have to think about and we're all really stressed, aren't we.<br>GP15-F-47	P17-F-36
	Everybody's understanding of percentages and statistics is very varied and for some people it's easy and for some people, understandably, it's not. And it's trying to kind of pitch that. And often you can tell sometimes when you're explaining things that it's just not appropriate…I just try and bring it back to, well, if there were 10 of you, one of you could end up having a heart attack and you might be one of the nine that never have a heart attack and so you take that statin and it makes no difference, or you could be that one person that is going to have a heart attack and you take the statin and then because of taking the statin, you don't have the heart attack.<br>GP2-F-38	*If you sort of put a number to it then, again, I suppose it's labelling it up really badly - 50 per cent might relapse. So, I think if you put it like you just said in a group of people, yeah, that would be better, for me anyway…probably because I think, and I know this sounds really silly, if you were to say you're probably this…in 40 per cent of people you'd think you're not on your own, that there's another 39 per cent stood behind you. There's 39 stood there with you.*<br>P6-F-52

	From a layperson's point of view, I would probably think proportions would work better, they can see themselves one in ten, so that's one person in ten, that's them, whilst ten per cent is the same, but that's just a bit more wordy, a bit more numbery, they may not fully understand it.<br>GP11-M-40	*It's obvious that people have a different way of learning, don't they…but, I must admit, I can visualise things better by actually seeing them, I mean percentages and things but sometimes, yeah, an actual diagram I know a lot of people would…that would make it a lot easier and simple for people to understand.*<br>P13-F-58
	So to explain the score, I normally say that if they, so, 'cause it's a percentage, I normally say, if there was 100 people exactly the same as you, same age, gender, blood pressure, blood results, all those things, this number out of 100 would have some sort of heart or stroke even in the next ten years. And I say, the higher that is the more likely it is that at some point that person's going to be you, out of that 100. And I say what number we tend to think of as being low risk, and what we think of as being high risk, so if the number's over 20 per cent, then, you know, then one in five, is actually quite a significant number of people who end up with heart	*If somebody is saying to you low risk or high risk or medium risk if they're face to face then straightaway you can say, well, what do you mean by that? So you can get that backup and have that more information.*<br>P13-F-58

	disease, and that's why we want to do something about it, and try and reduce that risk down. GP14-F-45	
Prognostic models for depression recurrence in primary care	*So it's very difficult to explain risk, I mean, there's whole books written about it for clinicians and it's still difficult. And I think for mental health…it is even more difficult. Again, it's very patient-led, some people are ambivalent, others are keen on treatment, others are keen to avoid really, so I think in those situations, it's trying to identify what the patient wants, what kind of person they are, but also looking at their future risks, if you know that their risk is borderline now, you can often say, well, you know what, in two years, your risk will be this, so why don't we initiate treatments now. Which again for mental health would be more difficult, because when you've got finite parameters such as age, cholesterol values, blood pressures, it's very easy for me to predict a future risk, mental health, again, much more abstract.* GP17-M-35	*Mental health is such a personal, personal thing, it's something that's so hard to put a statistic on or do averages and things on because everything's so personal. I don't know, I don't think it's even ever scientifically possible to put an exact number on anything to do with anything like this, so I don't think it's even worth trying.* P10-M-29

	I think depression is one of those things that everybody is affected by it so differently, that I just think that we always have to be careful with mental health tools that there is an element of individualisation and personalisation available. I think it's difficult with risk stratification tools, isn't it, because you're trying to put in…input data and have an answer. Mental health is always a bit more grey than black and white, than some other medical problems, isn't it.<br>GP21-F-33	*No, none of these markings or anything like that, statisticians love them, but we're talking about the mind here, we're not talking about an extra two inches on your putt or a climb or something. We're talking about reassurance. If a person feels relaxed and trusts you and the treatment that you are jointly going to go through with them… In the environment I had, yeah, you know, you want the right equipment in the right place at the right time to work and that is to kill people. Yeah, you need to be analytical, very direct, there's no messing about. This is not, this is a soft science here.*<br>P9-M-75
	There's always a place for anything that's going to help patient care, but I suppose it's how you capture a lot of that, to me it feels like quite sort of soft information. So, I mean, some of it is quite clear, so as you say, talking about patients who've had episodes before, so there's maybe a few things that are quite obvious and would be quite clear, but I guess a lot of the other things tend to be, I don't know, I suppose a conversation and then just a feeling you get for rather than	*It almost makes it like, more digestible doesn't it? It makes it more similar to physical health conditions that people are more familiar with? Like quantifiable – almost like you can measure depression, you know? Like you can measure blood pressure or something like that. Like that might provide a source of comfort for people, rather than it being this intangible thing that's going on in your brain…It's actually, look, here's these figures about what goes on, not in a scary way, not*

	something that's easily quantifiable.<br>GP10-F-44	*like, one in three people get cancer, you know, not something like that, but it almost makes it less scary I think, because it's like, oh look, there's so many people that experience this, there's figures about it, and I can be one of those people, and that's normal.*<br>P16-F-24
	You've got to be able to fit it into a ten-minute consultation, but at the same time, if it's going to save the patient a relapse and more time in the long-run, then I guess that's the thing to be thinking about. So, I think it would be fine as long as it wasn't too onerous and you could actually get through it. And usually by the time you've got to that point and if you're thinking about weaning or stopping, you know the patient fairly well, you've got that rapport, you know the background and things, so there's a lot of stuff you don't need to necessarily cover in that consultation. So, like I say, if there weren't too many questions, then I think it would be manageable.<br>GP8-F-52	*For me, I would say definitely, yes, because I would say… To me, that would all be part of somebody saying, you know, we are looking at this holistically. We're not just, you know, bandaging you up and getting rid of you. We care about what might happen downstream…Where I would find it gets a little bit difficult is if somebody said you are high risk of relapse but then there were no actions in place to mitigate that.*<br>P8-M-57

	I think it's always, you know, the more information that we have, the better, you can make more educated decisions. And I suppose, ultimately, we're wanting to make sure that patients are safe and, therefore, if we could flag up the ones that are more likely to relapse, and obviously by relapse they're more likely to, you know, self-harm, suicide, risky behaviour. But also, you know, from a workload point of view, if it meant that the patients that…aren't likely to relapse, maybe…maybe we could be more confident in saying, you know, we'll leave the doors open, you're in control of follow-up and we don't need to keep checking in and doing medication reviews every three to four months if actually they're stable.<br>GP2-F-38	*I feel like that's the sort of knowledge I'd prefer to have than not have. Say if I've had a particularly rough…or if I'm a patient that's had a particularly bad stint of depression, and categorises the high relapse category, I'd want to know that information, just so that I'm aware it can happen and I can take that into account so that I don't get false hope to be shattered further, kind of thing. Because there's nothing worse than believing that something's going to cure all your problems and that it's just going to go away, and then it coming back immediately and you're not knowing that it could do that or why. But knowing that you're in a…you're potentially high relapse category, but if that does happen, it doesn't mean it's the end for you, there is more that can be done, there is more that can be helped, just that reassurance of, it might happen to you, it's highly likely to happen to you, but if/when it does, don't become discouraged*

		because it can be fought, things can improve still.<br>P20-M-25
